# Quality of human-computer interaction - results of a national usability survey of hospital-IT in Germany

**DOI:** 10.1186/1472-6947-11-69

**Published:** 2011-11-09

**Authors:** Bettina B Bundschuh, Raphael W Majeed, Thomas Bürkle, Klaus Kuhn, Ulrich Sax, Christof Seggewies, Cornelia Vosseler, Rainer Röhrig

**Affiliations:** 1 Scientific Working Group "Clinical Information Systems", German Association for Medical Informatics, Biometry and Epidemiology (GMDS), Cologne, Germany; 2 Medical Informatics in Anesthesiology and Intensive Care Medicine, Justus Liebig-University, Giessen, Germany; 3 Department for Medical Informatics, Friedrich Alexander University, Nürnberg-Erlangen, Germany; 4 Department of medical statistics and epidemiology, TU München, Munich, Germany; 5 IT department, University medical center, Georg-August University, Göttingen, Germany; 6 IT department, University medical centre, Erlangen, Germany; 7 Vosseler Consulting, Mönchengladbach, Germany

## Abstract

**Background:**

Due to the increasing functionality of medical information systems, it is hard to imagine day to day work in hospitals without IT support. Therefore, the design of dialogues between humans and information systems is one of the most important issues to be addressed in health care. This survey presents an analysis of the current quality level of human-computer interaction of healthcare-IT in German hospitals, focused on the users' point of view.

**Methods:**

To evaluate the usability of clinical-IT according to the design principles of EN ISO 9241-10 the IsoMetrics Inventory, an assessment tool, was used. The focus of this paper has been put on suitability for task, training effort and conformity with user expectations, differentiated by information systems. Effectiveness has been evaluated with the focus on interoperability and functionality of different IT systems.

**Results:**

4521 persons from 371 hospitals visited the start page of the study, while 1003 persons from 158 hospitals completed the questionnaire. The results show relevant variations between different information systems.

**Conclusions:**

Specialised information systems with defined functionality received better assessments than clinical information systems in general. This could be attributed to the improved customisation of these specialised systems for specific working environments. The results can be used as reference data for evaluation and benchmarking of human computer engineering in clinical health IT context for future studies.

## Background

The aim of clinical-IT systems is to support the staff in high quality and cost-efficient patient care [1]. It is important to provide the appropriate information, at the appropriate location, to the appropriate individuals and in appropriate time [2]. The added benefit of information technology (IT) in hospitals has steadily grown during the last years due to increasing functionalities and penetration of medical processes. At present IT-systems are getting continuously more complex. Different studies have shown that users adjustments are associated with technology use and innovation acceptance [3,4]. A positive user attitude towards IT, IT-friendly environment and good communication can have beneficial influence on the system adoption [5]. Therefore, usability and especially the design of dialogues between humans and information systems is one of the most important issues to enable IT in health care [6]. The usability of a product is considered as a precondition of the usefulness of an application [7]. Different studies indicate the absence of usability is one of the main reasons for problems with IT implementations in hospitals [6,8-12]. The literature describes mostly single projects to analyse usability, for example just for one distinct/single information system or module [13-20].

The subject of the study presented here was to understand the current state of quality of human-computer interaction in the context of a national survey of usability (Usabil-IT) for hospital IT in Germany and to provide a descriptive picture of the present situation. The evaluation focused on effectivity and efficiency [21] as well as software ergonomics of deployed clinical-IT systems.

Different studies evaluating hospital IT show that usability is analysed best by targeting day to day users of IT-systems [18,22]. Therefore, the survey focused on participants using IT in day to day hospital work and demonstrates evaluation from different users' perspectives. The aim of the evaluation was also to identify starting points for developing the usability of the IT systems and for further, more detailed evaluation processes in the future.

## Methods

### Study design

Due to the fact that the approach of the study was to get an overview of the current situation of usability of clinical-IT in Germany, all IT system users of all systems had the opportunity to evaluate the usability of their primarily used IT-system. Therefore, the management of hospitals in Germany were contacted via e-mail and invited to participate in the survey with their clinical staff. The link to the start page of the survey could be forwarded to the employees. This desired open recruitment with the so called 'snowball-technique' produces a response bias, which has to be accepted from the outset [23]. On the one hand it offers a potentially wide reach and economy of administration, but on the other hand it also includes a tendency for self-selection-bias and relatively low response rates [24]. The variable of interest and the willingness to participate plays an important role in this process [25]. To mitigate the effects of the non-response bias in this kind of internet-survey is not feasible.

The survey was initiated by the scientific working group "Clinical Information Systems" of the German Association for Medical Informatics, Biometry and Epidemiology (GMDS) and by the Justus-Liebig-University Gießen (Department of Medical Informatics in Anaesthesia and Intensive Care).

The main focus of this survey was the investigation of human-computer interaction, which is defined by the ISO standard EN ISO 9241-10 [26,27]. The standard includes seven ergonomic principles (table 1). This part of the study is based on the IsoMetrics inventory, an assessment tool for these principles [22,28]. The IsoMetrics inventory provides empirically proved internal and external validity as well as the reliability of the results. Due to IsoMetrics' broad extent, the Delphi-Method [29,30] was used to truncate the questionnaire to the three principles most relevant for clinical-IT. The modified electronic questionnaires reliability was examined in a pre-test in two hospitals.

**Table 1:** Dialogue Principles according to ISO 9241-10 [22,49]

Dialogue Principles	Definition
Suitability for the task	A dialogue is suitable, if it helps the user to complete their tasks effectively and efficiently. Only those parts of the software are presented, which are necessary to fulfil the task.

Self-descriptiveness	A dialogue is self-descriptive, if every step is understandable in an intuitive way, or, in case of mistakes supported by immediate feedback. Further, an adequate support should be offered on demand.

Controllability	A dialogue is controllable, if the user is able to start the sequence and influence its direction as well as speed until they reach their aim.

Conformity with users expectations	A dialogue conforms with users expectations, if it is consistent, complying with the characteristics of the user, e.g. taking their knowledge in their special working area into account, likewise their education and experience as well as general acknowledged conventions.

Error tolerance	A dialogue is error tolerant, if the intended deliverable is reached with no or just minimal additional effort, despite of obvious faulty steering or wrong input.

Suitability for individualization	A dialogue is suitable for individualization, if the system allows customizing according to the task as well as regarding the individual capabilities and preferences of a user.

Suitability for learning	A dialogue supports suitability of learning, if the user is accompanied through different states of their learning process and the effort for learning is as low as possible.

To survey software effectiveness in addition of human-computer interaction, experts of the GMDS working group "Clinical Information Systems" developed questions focusing on functionality and interoperability in order to measure the effectiveness of clinical information systems (listed in table 2). For all items, closed questions allowed answers on a 5-point Likert scale [31]. With regard to human-computer interaction, three main evaluation criteria were examined: suitability for the task with 15 items, suitability for learning with 8 items and conformity with user expectations with 8 items. From the part of the study which focused on effectiveness 6 items on interoperability and functionality of IT Systems are presented in this paper. For each criterion reliability was verified using Cronbach's alpha, a measure for the internal consistency of a test score for a sample of examinees [32]. Criteria with values of 0.7 or higher are commonly regarded as reliable.

**Table 2:** Definitions of the different evaluated systems

Abbreviations	Systems	Definition
CIS	Clinical Information Systems	A collective or suite of applications that support medical work processes. It covers all the essential functions as a central computer system and is distinguished from specialised systems.

RIS PACS	Radiology Information Systems Picture Archive and Communication Systems	System for documentation and administration in radiology department. Captures digital images of all modalities, archives and communicates them.

LIS	Laboratory Information Systems	Software, which receives, processes and stores information generated by medical laboratory processes, incl. microbiology, pathology, e.g.

PDMS	Patient Data Management Systems	Provides patient-related information for use on Intensive Care Units.

AIMS	Anesthesia Information Management Systems	Supports the peroperative workflow and documentation.

ORIS	Operating Room Information Systems	Software, which supports the organisation in operating rooms.

AIS	Administrative Information Systems	Service which enhances the administrative operations.

Pharmacy IS	Pharmacy Information Systems	Offers supervision and inputs on the use of medication in hospital and pharmacy.

SRS	Staff Roster Systems	Manages staff, locations and rosters.

Med. Contr.	information systems for medical controlling	Medical Controlling is a staff position in German Hospitals for economic analysis and monitoring of the structure, process and outcome quality of medical service processes (especially financial controlling of the reimbursement in DRG-System). The system supports these working processes.

Other		Systems that were not evaluated by a large number of participants.

### Subjects and setting

Survey participants were asked specifically which IT system they mainly use. The evaluation of software ergonomics and effectiveness focused on the chosen IT-system, which was clearly defined for the respondents. Participants without direct IT contact (e.g. management) were excluded from this part of the survey.

Software ergonomics are always to be evaluated in context of task and situation. Therefore, the comparison of the results with data from other studies which focus on the same topic are important. Furthermore reference data for WinWord (Microsoft, USA) and SAP R/3 (SAP, Germany) of other IsoMetrics studies were integrated in the figures for comparison [22,33]. Although these references were gained in different studies with different collectives, the standardization of the IsoMetrics inventory allows comparison of the results and offers some sort of framework.

The reference line for Word processor and WinWord in general can be considered as gold standard for a software product with a clearly defined and narrow purpose; in contrast to SAP R/3 which sets a worldwide standard with its broad range of uses. These two references are considered as comparison data by the authors of the IsoMetrics inventory. Both have achieved a significant level of maturity and widespread use through a long development history.

### Data acquisition and data analysis

**Figure 1:**
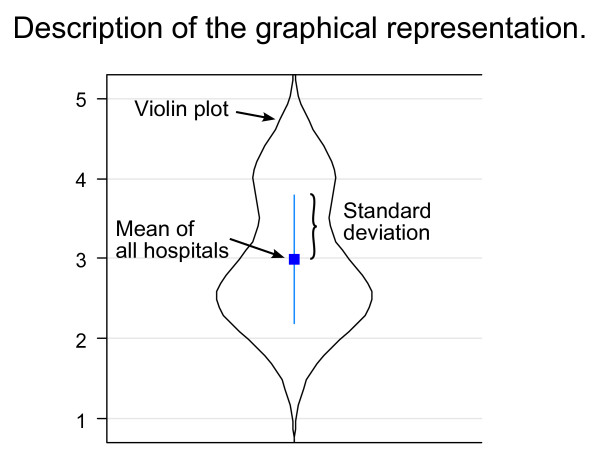
**Graphical presentation of the results**.

The survey data was acquired with the help of an online survey portal http://www.onlineforschung.org and analyzed using SPSS (V17.0.0, SPSS Inc). Questionnaires with incomplete IsoMetrics items were eliminated. Mean and standard deviation (SD) were calculated for each hospital and category of information system. Within figures mean values are displayed as blue quadrates and standard deviations as blue vertical lines. Added violin plots [34] show the relative frequency of the data at different values (Figure 1). Mentioned reference values for IsoMetrics figures are displayed as blue line (WinWord) and red line (SAP/R3).

## Results

### Study coverage data

In May 2009, the management of 2181 hospitals (9020 persons) were contacted via e-mail and invited to participate in the survey with their clinic staff. 4521 persons from 371 hospitals visited the start page of the survey, 1890 dropouts within the first page of the questionnaire were registered. Finally 1003 persons from 158 hospitals completed the entire questionnaire. The comparison of the sample characteristics with those of the population, statistics and data of the Federal Statistical Office in Germany [35,36], shows that the study is not representative. This is due to the open recruitment which does not allow a controlled population. Therefore a non-responder analysis was not feasible and definitive conclusions are not possible.

For the analysis presented in this paper only questionnaires were used, which had completed the entire software ergonomic questions. The sample characteristics are shown in the table 3.

**Table 3:** Sample characteristics

Occupation	participants	%
clinicians (physicians, nurses and allied health personnel)	658	65
non-bed-side medical personnel (radiologists, labratory)	73	7
administrative duties	127	13
IT-employees	41	4
IT-management	74	7
hospital management	32	3

Sum	1005	100

**IT-System**	**participants**	**%**

CIS	558	56
RIS/PACS	35	3
LIS	42	4
PDMS	27	3
AIMS	33	3
ORIS	25	2
Administr. IS	31	3
Pharmacy IS	14	1
Staff Roster	69	7
Med. Contr.	21	2
Other	112	11
no answer	38	4

Sum	1005	100

**Age**	**participants**	**%**

< 20 years	6	1
21 - 30 years	148	15
31 - 40 years	304	30
41 - 50 years	342	34
51 - 60 years	175	17
> 60 years	30	3

Sum	1005	100

**Sex**	**participants**	**%**

women	404	40
men	601	60

Sum	1005	100

**Work experience**	**participants**	**%**

< 6 months	15	1
6 months - 1 year	26	3
1 year - 5 years	149	15
5 years - 10 years	173	17
more than 10 years	642	64

Sum	1005	100

**Organizing institution**	**participants**	**%**

public	105	10
private	206	20
noncommercial	692	69
no answer	2	0,2

Sum	1005	100

**Number of beds**	**participants**	**%**

< 200	99	9,9
200-799	479	47,7
> 800	424	42,2
no answer	3	0,3

Sum	1005	100

### Study findings

The results show relevant variations between the different information systems, as well as a large standard deviation. Cronbach's alpha results for suitability for tasks recieved a = 0,944, for suitability for learning a = 0,851, for conformity with user expectations a = 0,883.

**Figure 2:**
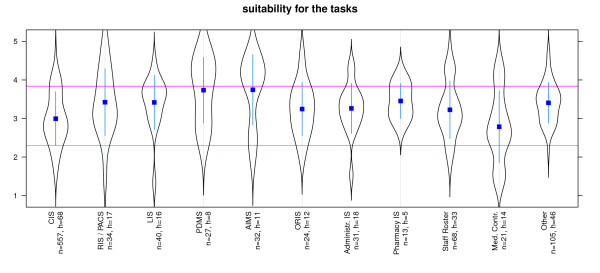
**Software ergonomics: 'suitability for the task' differentiated by systems**. n = calculated questionnaires, h = included hospitals (5 = good, 1 = poor) blue line: WinWord 2003, red SAP R3 [22,34]; Reliabiltiy (Cronbachs alpha) = 0,944 (pretest = 0,944).

**Figure 3:**
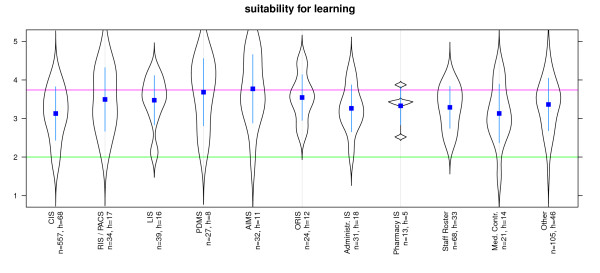
**Software ergonomics: 'suitability for learning' differentiated by systems**. n = calculated questionnaires, h = included hospitals (5 = good, 1 = poor) blue line: WinWord 2003, red SAP R3 [22,34]; Reliability (Cronbachs alpha) = 0,851 (pretest = 0,797).

**Figure 4:**
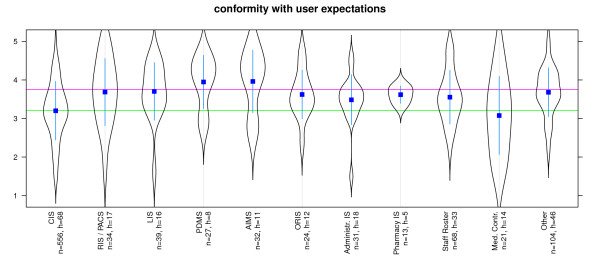
**Software ergonomics: 'conformity with users' expectations' differentiated by systems**. n = calculated questionnaires, h = included hospitals (5 = good, 1 = poor) blue line: WinWord 2003, red SAP R3 [22,34]; Reliability (Cronbachs alpha) = 0,883 (pretest = 0,891).

Suitability for tasks was best for AIMS, PDMS, Pharmacy IS, LIS and RIS/PACS. Systems for Staff Rosters, Clinical Information Systems and Medical Controlling received a poorer evaluation (Figure 2). Suitability for learning was best for AIMS, PDMS, LIS and ORIS. Systems for Medical Controlling, ERP and Clinical Information Systems received a poorer evaluation (Figure 3). Conformity with user expectations was best for AIMS, PDMS, Pharmacy IS, LIS and RIS/PACS. However, Administrative Information systems, Clinical Information System and Systems for Medical Controlling received lower ratings (Figure 4).

Most systems received similar results for all three dimensions, with the exception of a major variation within ORIS, displaying good conformity with user expectations and suitability for learning, with a lesser conformity in suitability for the tasks. Overall, highly specialised information systems with narrow and well defined functionality such as AIMS, PDMS, Pharmacy IS, LIS, RIS/PACS received better evaluations than possibly less individually customised Clinical Information Systems in general. The poor values for the Medical Controlling systems in all three tests are particularly noteworthy.

**Figure 5:**
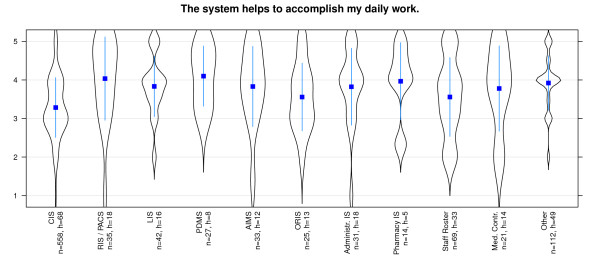
**Effectiveness/Functionality: 'coping of routine work' differentiated by systems**. n = calculated questionnaires, h = included hospitals (5 = good, 1 = poor).

**Figure 6:**
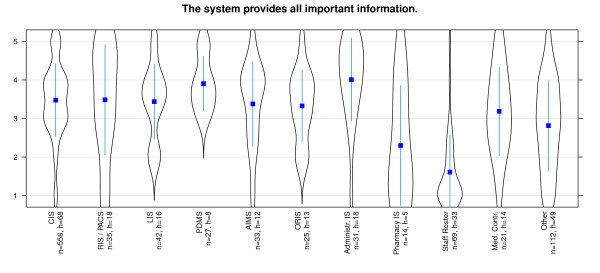
**Effectiveness/Functionality: 'presentation of all important information' differentiated by systems**. n = calculated questionnaires, h = included hospitals (5 = good, 1 = poor).

**Figure 7:**
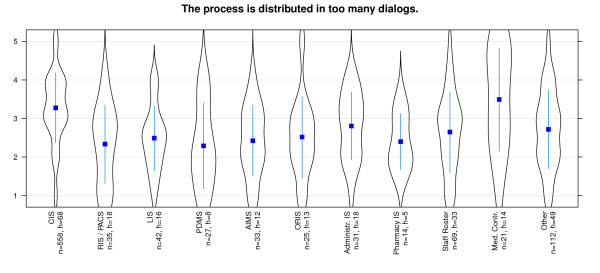
**Effectiveness/Functionality: 'too many different kind of modules' differentiated by systems**. n = calculated questionnaires, h = included hospitals (5 = good, 1 = poor).

The results of the study show that effectiveness in general is evaluated best for PDMS and RIS, while Pharmacy IS received poor results. Most systems have a wide spread distribution of values - especially AIMS, Medical Controlling System and Clinical Information System are conspicuous in this aspect. Coping with routine work is supported best by PDMS and RIS and worst with Clinical Information System (Figure 5). It strikes us, that this issue is rated with positive values in all systems. All important information is presented best with Administrative Information systems and PDMS (Figure 6). Great differences among the diverse systems are presented here. Bad results are achieved by Pharmacy IS and Staff Roster Systems. Especially users of Medical Controlling and Clinical Information systems affirm that there are too many different modules in use - in total contrast to Pharmacy information system users who deny this (Figure 7).

**Figure 8:**
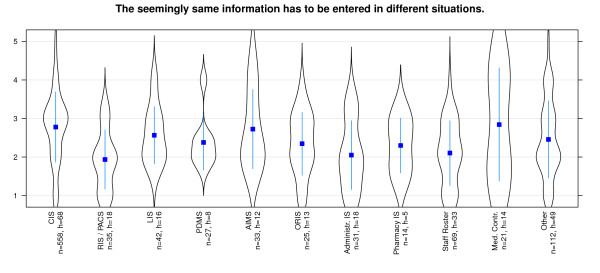
**Effectiveness/Interoperability: 'same information has to been added at different places' differentiated by systems**. n = calculated questionnaires, h = included hospitals (5 = good, 1 = poor).

**Figure 9:**
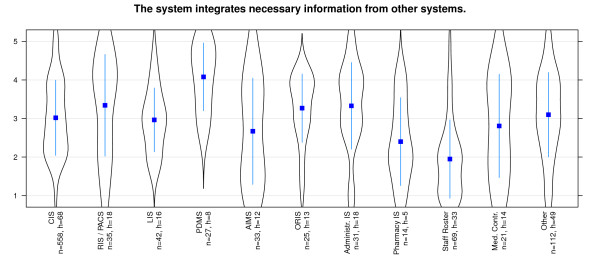
**Effectiveness/Interoperability: 'Data transfer between the systems' differentiated by systems**. n = calculated questionnaires, h = included hospitals (5 = good, 1 = poor).

**Figure 10:**
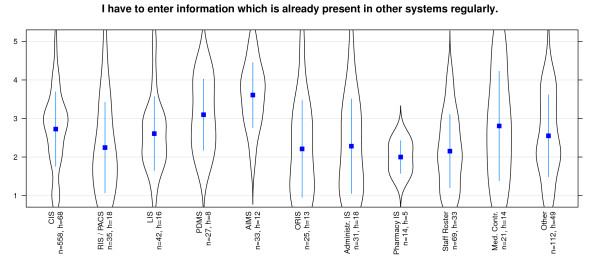
**Effectiveness/Interoperability: 'data requirement which has already been entered' differentiated by systems**. n = calculated questionnaires, h = included hospitals (5 = good, 1 = poor).

That the *same information has to be added in different places *is denied for almost all systems - especially for RIS and Pharmacy information system (Figure 8). The *ability of automatic data transfers *between different systems is evaluated as quite non homogeneous. While PDMS seem to provide this capability, there are low values for staff roster systems and pharmacy systems (Figure 9). The *requirement to input data*, which already has been entered somewhere else, is not necessary for most of the systems. Users of staff roster systems and pharmacy systems in particular score negative on this statement (Figure 10). In contrast, PDMS and AIMS seem to require redundant data input. It is notable that most systems present a wide distribution of values here.

## Discussion

### Answers to study questions

The results of the study show some clear findings. Relevant variations between the different information systems related to the human-computer interaction are presented. The issue of this evaluation was to find out how user-friendly IT systems in German hospitals are and where potential improvements to the efficiency might be possible. Usability evaluation is as a critical dimension for assessing the quality of clinical-IT especially focusing on all end users [37-39].

Our results show, that all examined IT systems meet the demand to support working processes. This is in line with the results of the survey by Fähling et. al.[6]. PDMS and RIS obtain the largest approval while Clinical Information systems perform poorly, although with equal distribution. This might be explained by the wide spectra of use of Clinical Information Systems, while PDMS and RIS are high specialised systems with clearly defined working processes. The low results for Staff Roster Systems in presentation of all important information might be explained by bad availability of data or non-functioning interoperability. Redundant data input is not totally avoidable by any examined IT system, but RIS and Administrative Information Systems seem to handle the problem best. This could be due to the fact that Administrative Information Systems keep record of all primary relevant data and communicate to all other clinical-IT systems. Data transfer is most effective with PDMS, which could be explained by the necessity to consolidate all important data. PDMS has typically high requirements on data integration. Functioning communication and interaction between the systems affects effectiveness immensely. Presenting information to the appropriate person, at the appropriate time in the appropriate display is an important aim of IT in medical fields [2]. Therefore, it is essential that data is transferred from other systems. The reason for the wide spread distribution of especially AIMS and Clinical information systems might be found in considerable quality differences of singular IT products and their implementation within clinics. In some cases needed functions are not implemented, while others are not used by the end users [40]. Health system usability is a significant factor in the implementation success [12]. Moreover, specialist systems in single fields with well-defined and structured usage patterns do better than those with a broad application area. It can be concluded that optimised adaptation to working environments and workflows of those specialised information systems result in optimised human-computer interaction. Most studies on usability of specialised IT-systems show positive evaluation [10,14,18,19]. In contrast, it seems to be much more complicated to design a good human-computer interface in the context of the many and varied tasks of a more generic clinical information system. An example is the different evaluation from users of AIMS and ORIS. AIMS are used in a very structured process by a limited user group. Its users undertake mainly documentation and information tasks. In contrast, the requirements of ORIS are much more complex. In operating theatre planning, much different data of patients, departments, personnel of different professions and specialties, theatre capacity, material and personnel resources have to be taken into account. In addition, documentation isn't generated contemporaneously and its generation cannot be assisted by automatic data capture, as is the case with AIMS. Ash et. al. could show that bad usability has negative influence on the work quality [38]. Efficiency regards the needed effort to achieve certain objectives. The aim of software ergonomics is to analyze and evaluate the usability of user interfaces of interactive computer applications [21]. Results of the best specialised systems (AIMS, PDMS, RIS, LIS, Pharmacy IS) are comparable to common specialised systems like Word processing software [22] or in the medical field e.g. a decision support system for antimicrobial therapy [14]. But even Clinical Information Systems, with a broad application area, are more suitable for the tasks and for learning and at least equal in conformity with user expectations compared to SAP R/3 [22]. In general, all three aspects "Suitability for the task", "Self-descriptiveness" and "Conformity with users expectations", which have been evaluated in this study for the different clinical-IT systems as basis for good software ergonomic, were rated positive and clearly better than reference data for SAP R/3.

The unexpectedly poor results of information systems for Medical Controlling could also be the result of more demanding users compared to other fields. At the end of the day, efficient oversight of diagnostic related groups (DRG) and consequently the accounting for the patients requires access to all available data. Thus maximal interoperability is needed for a good suitability for tasks. However, the usability of systems for medical controlling should be a focus for further research.

### Strengths and limitations of this study

Compared to the number of 2181 hospitals which received our questionnaire, the actual response rate of only 158 hospitals was less than expected, although higher than in similar studies [19,41]. While the response rate seems quite low, it is acceptable for nondirectional online surveys. Additionally, our dropout rate was much lower than usual for online questionnaires [42].

Despite the low response rate and potential bias in recruiting participants the survey shows some clear trends. Finally, multivariate analysis could not be performed as planned, because of the low number of participants in many subgroups. The large standard deviation is due to the fact that different clinics with different specialties and activities were examined and expertise among participants and software varied. Consequently, the results do not allow conclusions about individual products, but can permit statements about product groups. Further studies are needed to establish this kind of evaluation practice, as well as to get more detailed results and analysis of subgroups.

## Conclusions

This paper dealt with an online-questionnaire study about usability of clinical-IT in Germany. A summary is provided in table 4. The evaluation focused on IT-systems which are already in everyday use in hospitals as most of the studies reviewed by Peute et al. in 2008 do [43]. Fähling et al. could show that IT adds a positive value proposition to the hospitals [6]. The study Usabil-IT acknowledges this value proposition from the users' point of view.

**Table 4:** Summary

What was already known on this topic	What this study added to our knowledge
• Today hospitals depend on an effective and efficient IT environment to manage the complex requirements in every day work. It is known that IT adds an important value proposition to the hospitals. • Usability evaluations are necessary to estimate the quality of interactive IT-systems in clinical environment, while a lack of usability is one of the main reasons for problems with IT implementations. • System adaption depends on the users' attitude, which is influenced by the status of usability. Therefore it is important to evaluate not just from the experts' point of view, but also to pay attention to the direct users of clinical-IT.	• The survey offers the largest national usability data base of Hospital-IT in Germany so far. It allows the comparison of various clinical-IT systems and demonstrates evaluation from different user perspectives. • The findings of the study show that software ergonomic of clinical-IT is in the range of standard software, while specialised systems are more likely to receive better results than general systems. The effectiveness results show that all systems support the working processes, but also points out the differences between the diverse systems. • The study reveals several starting points for further studies • The available results of Usabil-IT provide an insight into the current state of usability of clinical IT. They can be used for developing and evaluation processes in the future.

Despite the above mentioned limitations, the largest data base with more than 1000 fully answered questionnaires was compiled. The study shows clear trends in the status of usability of clinical-IT in German hospitals. The results are self-consistent and show that usability is a very important issue considering clinic IT acceptance and usage [3-5,44]. Therefore, they can be used as reference data for evaluation and benchmarking of user-oriented software engineering for hospital health care IT, which is relevant for the development of hospital IT systems and therefore for clinical practice too. Furthermore the study Usabil-IT covered most of the key performance indicators to benchmark hospital information systems published by Hübner-Bloder and Ammenwerth end of 2009 [45].

The software ergonomics are mainly evaluated positively which is in line with the results of other studies [19,41,46]. The generally acceptable results for software ergonomics in this study do not support the statement by Fähling et al. who emphasises that a lack of usability is one of the main reasons for problems in assignments of clinical-IT.

The usability has been measured in different ways, in this paper the results were presented for software ergonomics and effectiveness differentiated by IT systems. The user satisfaction scores were generally positive with some differences between the systems. This basically positive feedback is related to the results of other studies [19,47,48]. It is shown that almost all clinical-IT systems support effective working processes; a gain of added value by hospital IT can be concluded. However, it is notable that most systems have a widespread distribution of values in this aspect. Thus, considerable quality differences of products and implementations in different clinics were detected. Most strikingly, specialised information systems result in more optimised human-computer interaction, while general systems, which have to solve many and varied tasks, get poorer evaluation. This implies that higher integration needs and further improvement for general systems in daily practice are necessary. Also the unexpectedly poor results of information systems for Medical Controlling are astonishing and should be a focus for further research.

## Competing interests

The presented results are part of the national survey "Usabil-IT", which was financially supported by the German Association of the Healthcare IT Industry (VHitG). The VHitG did not have any influence on the results or the conclusions of the survey. The survey follows the international code for market and social research (ICC/ESOMAR).

## Authors' contributions

BBB carried out the study conception and design and performed the acquisition and interpretation of data and drafted the manuscript. RWM performed the statistical analysis, constructed the graphics and drafted the articles technical parts. TB, KK, US, CS and CV were involved in conception of the questionnaire as well as in the interpretation and discussion of the results. RR conceived of the study and participated in the design of the study. He was also involved in drafting the article. All authors read and approved the final manuscript.

## Pre-publication history

The pre-publication history for this paper can be accessed here:

http://www.biomedcentral.com/1472-6947/11/69/prepub
